# Prevalence of *Helicobacter pylori* in dyspeptic patients at a tertiary hospital in a low resource setting

**DOI:** 10.1186/s13104-015-1184-y

**Published:** 2015-06-23

**Authors:** Michael Oling, J Odongo, O Kituuka, M Galukande

**Affiliations:** Department of Surgery, College of Health Sciences, Makerere University, P. O. Box 7072, Kampala, Uganda

**Keywords:** *H. pylori*, Gastritis, Endoscopy

## Abstract

**Background:**

More than half of the world’s population is infected with *Helicobacter pylori* (*H. pylori*), the primary cause of chronic gastritis. Chronic gastritis is associated with peptic ulcer and in advanced stages with an increased risk of developing gastric adenocarcinoma. In many developing countries access to upper gastrointestinal (UGI) endoscopy services is limited. As a result, many UGI diseases are treated empirically.

**Objective:**

To determine the prevalence of *H. pylori* in patients presenting with dyspepsia, and the mean time from onset of symptoms to performing an endoscopy examination.

**Methods:**

A cross sectional descriptive study conducted from 5th January to 30th April 2014. Adult patients with dyspepsia who were referred for UGI endoscopy were recruited consecutively. Questionnaires were used to collect data which were analyzed using STATA software. IRB approval was obtained.

**Results:**

In total, 111 participants’ data were analyzed. The F:M ratio was 1:1.4, mean age 43 years (SD = 16). The prevalence of *H. pylori* gastritis was 36%. The minimum time to endoscopy was 3 weeks, maximum 1,248 weeks and the mean time 57 weeks.

**Conclusion:**

The burden of *H. pylori* infection in patients with dyspepsia was high. Patients had prior empirical antibiotic therapy. Access to endoscopic services is limited.

## Background

Endoscopy is essential in the classification of the patient’s condition as organic or functional dyspepsia [1–5]. *Helicobacter pylori* is a gram negative microaerophilic bacterium that causes inflammation of the stomach, first isolated by Barry Marshall and Robin Warren in 1982. It is a highly prevalent infection in developing countries with poor socio-economic status and an etiologic agent of the majority of upper gastrointestinal (UGI) diseases associated with significant morbidity [6]. The regimens recommended by National institute of Clinical Excellence for *H. pylori* eradication are omeprazole, amoxicillin, and clarithromycin (OAC) for 10 days; bismuth subsalicylate, metronidazole, and tetracycline (BMT) for 14 days; and lansoprazole, amoxicillin, and clarithromycin (LAC) for 10–14 days of treatment. More than half of the world’s population is infected with *H. pylori*, the primary cause of chronic gastritis. Chronic gastritis is associated with peptic ulcer and in advanced stages with an increased risk of developing gastric adenocarcinoma [7]. In many developing countries facilities for UGI endoscopy are rare. As a result, the diagnosis of UGI diseases is carried out solely on clinical parameters in most cases. Incorrect/delayed diagnoses, and subsequent ineffective management, results in increased morbidity, economic loss to the client, and even death especially in patients with malignancy [4]. The objective of this study therefore was to determine the prevalence of *H. pylori* associated gastritis among patients with dyspepsia and to determine the mean time from onset of symptoms to endoscopic procedure.

## Methods

This was a cross-sectional descriptive study of patients with dyspepsia that presented to a tertiary hospital for UGI Endoscopy from 5th January to 30th April 2014.

Mulago National Referral Hospital (MNRH) doubles as Makerere University Teaching Hospital and therefore receives patients from all over the Country as well as the neighboring countries for expert management. MNRH is located in Kampala the capital of Uganda. Patients with dyspepsia present to the gastroenterology clinic. Further management depends on the decision made by the attending doctor. Some patients are sent for endoscopy, others are started on empirical treatment.

All patients who presented with dyspepsia during the study period and met the inclusion criteria were recruited consecutively upon signing a written informed consent.

The inclusion criterion was patients presenting with undiagnosed dyspeptic symptoms and age 18 years and above.

*Dyspepsia symptoms included* One or more of the following symptoms; postprandial fullness (termed postprandial distress syndrome); early satiation (meaning inability to finish a normal sized meal or postprandial fullness) and Epigastric pain or burning epigastric pain (termed epigastric pain syndrome).

*Exclusion criteria* Patients with alarm symptoms (weight loss, haematemesis, persistent vomiting) were excluded from this study.

Patient preparation involved 6 h of fasting. In conscious patients, a topical anesthetic xylocaine 5% was sprayed into the oropharynx to numb the gag reflex. Sedation with intravenous midazolam 0.1 mg/kg was used at the discretion of the attendant.

Endoscopic evaluation of patients was carried out using a fibre optic gastro-duodenoscope Olympus and following standard procedures. Instrument sterilization was done using a routine technique of cleaning the instrument with cetrimide, 70% alcohol, glutaraldehyde (Cidex®) and later running equipment in distilled water for up to 30 min in between endoscopic sessions. Patients were placed in the left lateral decubitus with pulse oximetry monitoring of their vital sign by the anaesthetist. All anatomic regions of the esophagus, stomach, first and second parts of the duodenum whenever possible were examined and endoscopic impressions noted. Pinch mucosal biopsies for histopathological diagnoses and *H. pylori* detection were obtained from the antrum, stomach and suspicious areas for all cases.

The diagnoses conformed to standards as reflected in the atlas of gastrointestinal endoscopy [8].

The histopathological diagnosis of *H. pylori* infection in biopsy specimen was done using the Modified Giemsa stain which is easy to interpret, inexpensive, and takes about 5 min to perform, and rarely requires repeat stains. It has a sensitivity of 98% and specificity of 90% [9].

### Study variables

The social demographic factors were: age, sex, occupation, marital status, home address, and level of education. Others included: alcohol consumption, cigarette smoking, and use of non steroidal anti-inflammatory drugs, proton pump inhibitors, and antibiotics. Also included were clinical variables: postprandial fullness, early satiation, epigastric pain.

The dependent variables were microscopic diagnoses (histologically confirmed *H. pylori* associated gastritis), changes of inflammation, dysplasia or metaplasia, cancer of the upper gastrointestinal tract and macroscopic diagnosis (endoscopic diagnoses) including but not limited to duodenitis, peptic ulcer disease (PUD), gastroesophageal reflux disease (GERD) using savory miller grading.

### Data analysis

Data were analyzed using STATA version13. The results were presented as mean ± standard deviation for quantitative variables and number (percentages) for qualitative variables. Categorical variables were compared with Pearson’s Chi square. Significant *P* value was taken as <0.05 at 95% CI.

Gastritis was further classified into its various forms. The prevalence of *H. pylori* associated gastritis was calculated as total number of patients with *H. pylori* associated gastritis divided by the total number of study subjects and expressed as a percentage.

The topography/patterns of gastritis was documented as pan-gastritis or antral predominant, etc. and expressed as percentages out of the total cases of gastritis.

### Ethical considerations

Ethical approval was obtained from the Research and Ethics Committee of the College of Health Sciences of Makerere University. Written informed consent was obtained from all the participants.

## Results

**Figure 1:**
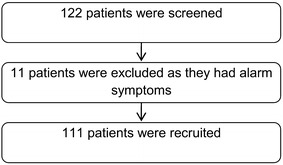
Recruitment flow chart.

We recruited 111 patients (see Figure 1) with a F:M ratio of 1:1.4. The mean age was 43 years (SD 16) and the median age was 40. All the participants were residing in Kampala an urban area. All the participants had formal education with the majority having attained a tertiary level of education 52/111 (47%).

### Risks factors

There were nine smokers, smoking an average of two stick a day, over 52–502 week duration. However one participant smoked four sticks a day.

The majority 97/111 (87%) of study participants did not consume spirits. Of the 14 who took spirits, 8 (57%) took about a liter a week, and 2 (14%) consumed about 3 L a week.

The majority of the study participants that consumed beer took about 2 L a week, except one male who reported consumption of about 8 L on average every week.

Two study participants reported wine consumption, about 0.5 L a week.

Nine participants, all above 55 years of age had been on NSAIDS for over 3 weeks.

Of the 34 participants who presented with post prandial fullness, 25 (73%) had it for 6 months or longer. Of the 25 participants who presented with early satiety, 20 (80%) had it for 6 months or longer. Of the 100 patients who presented with epigastric pain, 86 (86%) had it for 6 months or longer. Seven cases had heart burn as well as dyspepsia, 10/111 (9%) had a combination of all three symptoms of dyspepsia and 12/111 (11%) had a combination of two symptoms.

The overall prevalence of *H. pylori* associated gastritis was 40/111 (36%) at 95% CI (27–45).

Out of the 40 patients with *H. pylori* at histopathology, 21 (52.5%) were in a tertiary institution, or had attained a tertiary level of education, 31/40 (77.5%) had been taking PPIs at the time of endoscopy. The highest prevalence 22/40 (55%) was in the age category of less than or equal to 40 years and 17/40 (42.5%) had not been on antibiotics at the time of endoscopy. Out of the 76 patients who had moderate epigastric pain, 24 (32%) had *H. pylori* at histology. The majority [35/40 (87.5%)] cases of *H. pylori* were seen in biopsy specimen from the stomach (Table 1).

**Table 1:** *H. pylori’s* distribution by age groups, level of education, antibiotic use, severity of epigastric pain, early satiety, post prandial fullness, and endoscopic macroscopic findings

Variable	*H. pylorus* absence number (%)	*H. pylorus* presence number (%)	OR (95% CI)	p value
Age group in years				
≤40	34 (48)	22 (55)		
>40	37 (52)	18 (45)	0.75 (0.35–1.64)	0.472
Level of education				
Primary	11 (15)	5 (13)	Reference	
Secondary	29 (41)	14 (35)	1.06 (0.31–3.65)	0.924
Tertiary	31 (44)	21 (53)	1.49 (0.45–4.92)	0.512
Antibiotic use				
None	42 (59)	17 (43)	Reference	
≤2 weeks	25 (35)	22 (55)	2.17 (0.97–4.86)	0.058
>2 weeks	4 (6)	1 (3)	0.62 (0.06–5.93)	0.676
Severity of pain				
Mild	6 (9)	3 (8)	Reference	
Moderate	52 (81)	24 (65)	0.92 (0.21–4.01)	0.915
Severe	6 (9)	10 (27)	3.33 (0.60–18.54)	0.169
Macro findings				
Stomach	56 (82)	35 (88)		
Others	12 (18)	5 (13)	0.67 (0.22–2.05)	0.480
Early satiety				
Never	54 (76)	33 (82.50)		
Experienced	17 (24)	7 (18)	0.67 (0.25–1.80)	0.430
Postprandial fullness				
Never	46 (65)	31 (78)		
Experienced	25 (35)	9 (23)	0.53 (0.22–1.30)	0.166

The association of *H. pylori* and the above variables was not statistically significant [the p values were greater than 0.05 at 95%.

The minimum duration of symptoms before endoscopy was 3 weeks, and the maximum 1,248 weeks. The average time to endoscopy was 126 weeks, and the median 57 weeks.

**Figure 2:**
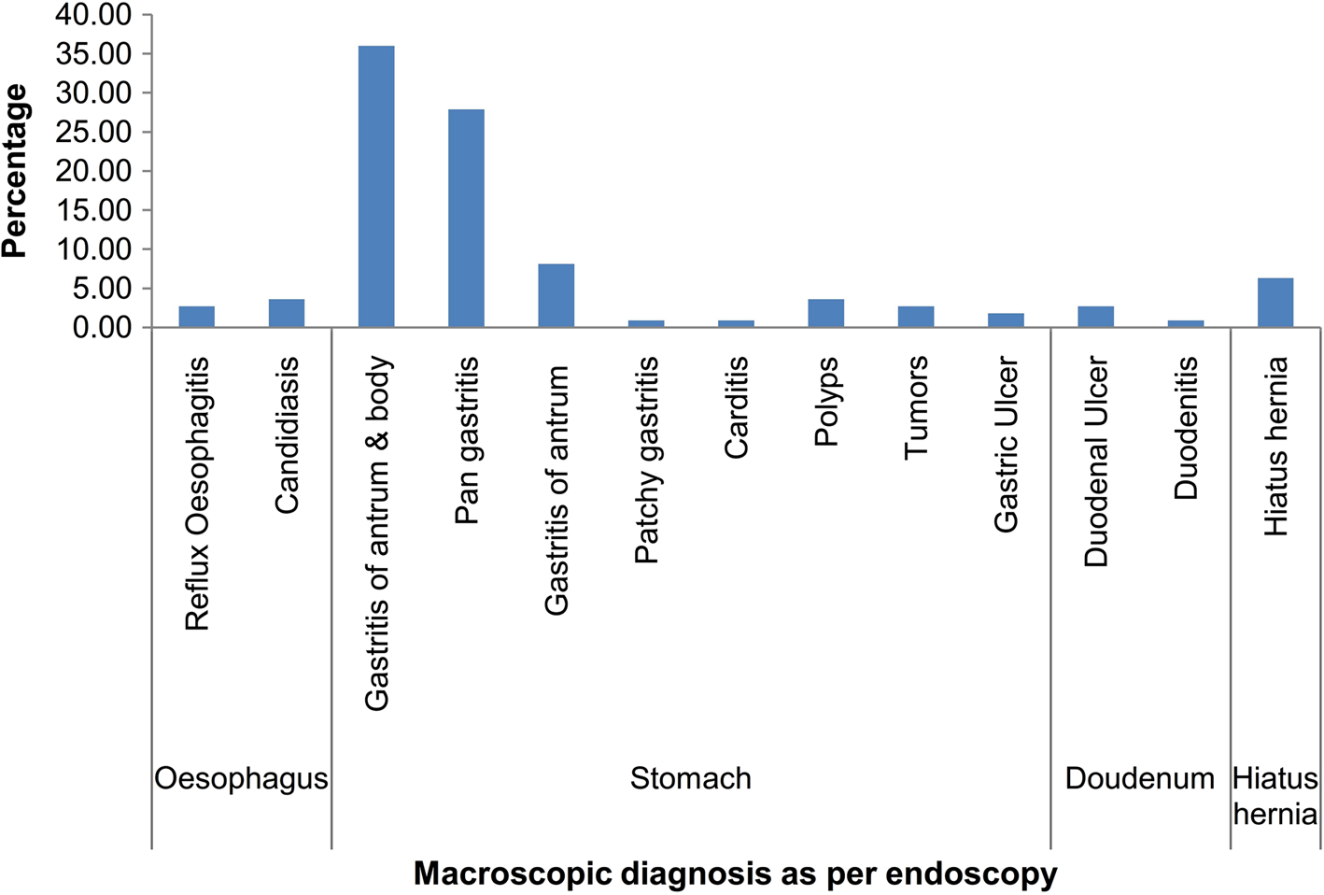
Distribution of macroscopic lesions by region.

The commonest diagnosis was gastritis 82/111 (74%). The commonest pattern of gastritis was moderate gastritis of the antrum and body 40/82 (49%) followed by moderate pan gastritis 24/82 (29%). Only 7/111 (6%) participants had severe disease (gastric ulcer, duodenal ulcer and tumors) see Figure 2.

The tumors were involving the gastro-jejunal junction and fundus and the other was in the cardia.

3 patients had normal findings at both endoscopy and histopathology.

There were 7/111 (6%) cases of hiatus hernia. Three of these were large and associated with reflux oesophagitis savory miller grade 1 at endoscopy.

The commonest histopathological diagnosis was chronic non active gastritis.

A total of 40/111 (36%) participants had *H. pylori* at histopathology.

The two cases of duodenal ulcer had *H. pylori* infection at histopathology.

One participant who had moderate gastritis of the antrum and body at endoscopy had adenocarcinoma in situ. The other case of adenocarcinoma had a mass involving the fundus and gastro esophageal junction.

The tumor that was involving the cardia turned out to be a moderately differentiated squamous cell carcinoma.

## Discussion

We set out to establish the prevalence of *H. pylori* gastritis among patients presenting for endoscopic examination.

We found that over 36% had *H. pylori* gastritis and that it took 57 weeks on average for the participants to access endoscopic services from the time of developing symptoms. *H. pylori* prevalence goes from less than 15% in some populations to virtually 100% depending on socio economic status and country development. In High income Countries exposure tends to occur later in life, which results in lower percentages in infected adults, an average of 20–30% of adults are infected by age of 50. In this study those ≤40 years was 55%, twice as much as stated for some High Income Countries. It is anticipated that the prevalence of *H. pylori* infection will decline as sanitary conditions improve and it is also a reflection of wide spread use of antibiotics [10].

*Helicobacter pylori* infection has been reported by several studies to be high in developing countries, and associated with low levels of education, low social economic status, and poor sanitation [4, 11].

NSAID use and *H. pylori* infection have a significant impact on endoscopic findings while presence of *H. pylori*, smoking and alcohol consumption are all associated with increased risk of developing chronic gastritis [11]. In this study the consumption of cigarettes was low however the consumption of alcohol was moderate.

The overall prevalence of *H. pylori* associated gastritis may be an under estimate since the majority of the participants had been on prior empirical treatment with antibiotics. However, this value is similar to one found by Wabinga et al. [12] in his retrospective study in 2002. In contrast, studies in 2012 in neighboring Kenya [13] reported a 52% prevalence of *H. pylori* in adults [14].

In Nigeria, a 41% prevalence was reported in Lagos state in 2008 [11]. Notably, all the above studies were carried out in an urban settings where there is congestion, perhaps sub optimal sanitary conditions but easy access to antibiotics compared to rural settings.

In the East Cape province of South Africa in 2008, 66.1% *H. pylori* prevalence was reported [15]. The East Cape Province is one of the poorest provinces of South Africa.

The majority of patients (close to 80%) had been on prior empirical treatment as opposed to the test and treat approach practiced in some countries with a low prevalence of *H. pylori*.

However close to half of the *H. pylori* infected adults had not been on antibiotics at the time of endoscopy. Also one patient had *H. pylori* despite taking eradications antibiotics for over 4 weeks prior to endoscopic examination. Perhaps a case of resistance to the regimen used.

The odds of *H. pylori* positivity were reduced in those who had taken antibiotics for more than 2 weeks. OR = 0.62 (0.06–5.93). A possible explanation is that eradication is likely achieved after 2 weeks of therapy.

The time from onset of symptoms to the endoscopic procedure was not normally distributed. The minimum duration of symptoms before endoscopy was 3 weeks, and the maximum 1,248 weeks. The average time to endoscopy was 125.6 weeks, and the median 57 weeks. This is the first study that looked at the time duration from onset of dyspeptic symptoms to the endoscopic procedure in Uganda. This reflects on the limitation to accessing endoscopic services. The findings suggest that the average time from onset of symptoms to the definitive diagnosis by endoscopy is about a year. This might be a long time, bearing in mind that *H. pylori* is a potent risk factor for malignancy [12]. In this study, a 37 years old presented with dyspepsia (without alarm symptoms), was found with moderate *H. pylori* gastritis of the antrum and body and adenocarcinoma in situ. Clinicians have to be aware that dyspepsia in the young could be cancer [16]. In a systematic review of 4,018 patients the use of alarm symptoms to select dyspeptic patients for endoscopy caused patients with early curable cancers to be overlooked [17].

The commonest macroscopic finding was gastritis similar to Kagimu et al. [12], Wabinga et al. in Uganda [12], Sang Thomas et al. [13] and Kimang et al. [14] In Kenya, Sang Thomas et al. found 4% normal findings at endoscopy and Kimangi et al. found that 100% of the study participants with dyspepsia had abnormal findings at endoscopy. In the Eastern Cape province S.A, 33.6% of the patients had functional dyspepsia [15]. Similarly, Abioudun et al. in Nigeria found that gastritis was the commonest finding, but also found a high incidence of *H. pylori* in the endoscopically normal study participants [18]. In contrast, in Italy, a case control study by Zagari et al. of 1,033 study participants found that ¾ were normal endoscopically. In his study, 93.4% of PUD patients had *H. pylori* at histology [19]. In Korea, Jung et al. and colleagues found that 40% of the study participants were normal endoscopically in 2012.

Despite the relatively high prevalence of *H. pylori* infection, only a few study participants had severe disease (PUD and malignancy). Two [2] participants had *H. pylori* associated duodenal ulcers, and three patients had malignancy. Sang Thomas et al. in Kenya found that 30% of his study participants had PUD with *H. pylori* and 73.6% of these had *H. pylori* [13] of the 82 patients with Gastritis 7 (8.5%] had severe gastritis at endoscopy and their histology confirmed *H. pylori* presence.

This study found that seven participants (6.3%) had hiatus hernia at endoscopy presented with dyspepsia plus heart burn. This is in contrast to the majority of the studies mentioned above that all had less than 2% of these cases and reflux oesophagitis.

### Study limitations

One major limitation of this study is that it was conducted in a hospital setting and this may not be a true representation of the prevalence of *H. pylori* among dyspeptics in the general population. A community study is therefore desirable. Study involving asymptomatic controls was desirable but because endoscopy is expensive and invasive, it was not possible to get study participants.

The study participants did not have an abdominal ultra sound done to exclude other causes of undiagnosed dyspepsia, pancreatitis and hepatobiliary disease, especially for those that had normal endoscopic findings.

There could have been recall bias as participants were required to remember when their symptoms started.

## Conclusion

The burden of *H. pylori* infection among dyspeptic patients was high. There is limited access to endoscopic services and widespread prior antibiotic empirical treatment. Gastritis is the commonest finding at endoscopy in patients presenting with dyspepsia.
